# Prescribed Performance Back-Stepping Tracking Control for a Class of High-Order Nonlinear Systems via a Disturbance Observer

**DOI:** 10.3390/e25010103

**Published:** 2023-01-04

**Authors:** Xinrui Tang, Haijun Jiang

**Affiliations:** College of Mathematics and System Science, Xinjiang University, Urumqi 830046, China

**Keywords:** input-to-state stability, disturbance observer, prescribed performance, back-stepping control, high-order systems

## Abstract

Due to the widespread presence of disturbances in practical engineering and widespread applications of high-order systems, this paper first pays attention to a class of high-order strict-feedback nonlinear systems subject to bounded disturbance and investigates the prescribed performance tracking control and anti-disturbance control problems. A novel composite control protocol using the technique of a disturbance observer—prescribed performance control—is designed using the back-stepping method. The disturbance observer is introduced for estimating and compensating for unknown disturbances in each step, and the prescribed performance specifications guarantee both transient and steady-state performance of the tracking error to improve the control performance and result in better disturbance rejection. Moreover, the technique of adding a power integrator is modified to tackle controller design problems for the high-order systems. The Lyapunov function method is utilized for rigorous stability analysis. It is revealed that while the control performance completely remains in the prescribed bound, all states in the closed-loop system are input-to-state stable, and the tracking error and the disturbances estimating error asymptotically converge to zero simultaneously. Then, the feasibility and effectiveness of the proposed control protocol are verified by a simulation result.

## 1. Introduction

Any practical system in life has different degrees of nonlinear properties. Since the Dutch meteorologist Lorenz opened the door to mankind’s understanding of the nonlinear world in the 1960s, the control problem of nonlinear systems has been in full swing [1,2,3,4]. There are many main research methods, such as differential geometry methods, passivity theory, Lyapunov stability theory, and so on. Due to the complexity and diversity of nonlinear systems, different methods are applicable to different problems.

In many practical control problems, one often needs to quantitatively characterize the impact of errors and disturbances in measurement elements or execution mechanisms in the system, so the stability of the forced system is the most fundamental issue in control theory. There are two approaches to describing the stability of forced systems with different ideas: one uses the operator theory technique. The input–output stability based on the small gain theorem can obtain great results in applying to a infinite network [5]. The other is the application of the state space approach. Sontag in [6] introduced the concept of input-to-state stability for the first time to systematically describe the stability of a forced system by the state space approach. This approach replaces the finite gain with a nonlinear gain function, resulting in fewer limitations, and the advantage of having multiple equivalent representations (e.g., Lyapunov-like description of the function) makes it more compatible with existing control theory. Nowadays, it has been widely used in neural networks [7], $H_{\infty }$ control [8], inverse optimal control [9], stochastic systems [10], time-delay systems [11], switching systems [12], discrete systems [13], etc.

Moreover, due to practical engineering needs, tracking control is often one of the main control objectives for nonlinear forced dynamic systems—for example, see [14,15,16] and references therein—which aims to make the system output asymptotically track our expected dynamic signal by applying inputs to the system.

Although there have been many related studies about tracking control, in order to better apply it to engineering systems, studies in the past decades started to be keen on solving the tracking control problem with external disturbances in the system. With the increasing requirements for control accuracy, many control methods have been proposed for systems with various disturbances and parameter uncertainties: for example, nonlinear $H_{\infty }$ control [8], sliding mode control [17], output regulation theory [18], and adaptive methods [19]. Although the above methods effectively attenuate or reject disturbances, the output regulation theory requires the derivative of the controller [20], whereas most other methods sacrifice nominal system performance when achieving robustness [21]. To avoid above effects, Nakao et al. proposed disturbance observer-based control (DOBC) in the late 1980s, which can estimate unknown disturbances that are difficult to be measured directly by sensors, and compensates for the equivalent disturbances in the feed-forward channel. Owing to its excellent interference rejection capability, DOBC has been widely used in various practical systems, such as servo systems [22] and robot systems [23]. Reference [24] proposed a method combining DOBC and sliding mode control to estimate the disturbance and attenuate it using a designed sliding surface. However, the tracking errors in the existing studies [25] do not reach asymptotic convergence. Subsequently, the back-stepping method was proposed [26]. This method decomposes a complex nonlinear system into subsystems and uses the introduction of virtual control law and the design of Lyapunov functions for each subsystem to complete the controller design for the entire main system. With the development of the back-stepping method, the construction of controllers have a systematic approach, which was then introduced in the literature [27] along with DOBC for the disturbed nonlinear problems.

Most existing tracking control problems have focused on solving stability problems without considering constraints on the transient performance of the system before reaching steady state, which is often limited by factors such as hardware and interaction with humans. More than a decade ago, Bechlioulis and Rovithakis first proposed prescribed performance control (PPC) in [28,29] for nonlinear simple input simple output (SISO) system and multi-input multi-output (MIMO) systems, where the transient and steady-state performance of the system can be constrained simultaneously using the performance function, and asymptotic tracking control is achieved. PPC is gradually being used to solve various control problems [30,31]. In [32], Chen and Yang introduced a novel performance function into PPC and developed a controller based on the back-stepping method to achieve tracking control with prescribed performance.

It should be noted that, on the one hand although the PPC approach allows the tracking error to be always kept within the prescribed constraint, it is still difficult to design a specific composite controller to simultaneously guarantee the prescribed performance and achieve tracking control if external disturbances are present in the system. Bai et al. only considered the prescribed performance tracking control problem for high-order nonlinear systems without and external disturbances [31]. On the other hand, most studies of control methods that introduce disturbance observers, to our knowledge, did not consider prescribed performance specifications [22,23,24,25]. In addition, it is also noted that there has been few previous studies dealing with the problem of high-order nonlinear systems. Chen et al. investigated an adaptive output feedback control law for first-order, unknown, pure-feedback nonlinear systems with external disturbances [32]. As far as we know, there has been no work so far considering simultaneous prescribed performance tracking control and anti-disturbance control problems for high-order nonlinear systems, which gave the motivation to carry out this work.

Inspired by this, a composite controller was designed for a class of high-order strict feedback systems with external disturbances to address the prescribed performance-tracking problem. Concretely, a disturbance observer is used to estimate and compensate for external disturbances; a prescribed performance function and a transformation function are used to convert the original prescribed performance tracking control into an unconstrained system with the same stability; and finally, the DOB technique and a back-stepping method incorporating adding a power-integrator technique for dealing with high-order systems problems are used in the controller. This control scheme ensures the prescribed transient and steady-state behaviors of the tracking errors while enabling a high-order nonlinear system with stronger disturbance rejection. This article has the following contributions relative to existing results:

(1) The proposed composite controller solves the output tracking problem of a class of high-order nonlinear systems, where the system states are stabilized and the tracking error converges to zero.
(2) Differently from the methods designed to attenuate disturbances to a specified area [33,34], the error systems of nonvanishing disturbance estimating converge to zero. That is, this control scheme can eliminate the affect of disturbances on output.
(3) Without the external disturbances, the nominal control performance of the proposed protocol remained.
(4) Unlike the previous results in [30,35], the performance indices of the system regarding transient steady-state behavior are not only allowed to evolve within a prescribed bound, but also guarantee zero steady-state output tracking error.

The rest of this article is organized as follows. In Section 2, the problem formulation and preliminaries are given. In Section 3, the composite control protocol is constructed by utilizing back-stepping technique, and the stability and the prescribed performance are analyzed. In Section 4, an example is given to show the effectiveness. A short conclusion is given in Section 5.

## 2. Problem Formulation and Preliminaries

### 2.1. Problem Formulation

Following notation is used throughout the paper. For a given vector $x={\left(x_{1},\ldots ,x_{n}\right)}^{T},$$∥x∥={\left(x_{1}^{2}+\cdots +x_{n}^{2}\right)}^{\frac{1}{2}}$ is the Euclidean norm of vector *x*. $\;{\bar{x}}_{i}={\left(x_{1},\ldots ,x_{i}\right)}^{T},i=1,\ldots ,n.$

Consider a class of high-order strict-feedback nonlinear systems modeled by

(1) $$\left\{\begin{array}{c} {\dot{x}}_{1}\left(t\right)=x_{2}^{p_{1}}\left(t\right)+\phi_{1}\left({\bar{x}}_{1}\left(t\right)\right)+d_{1}\left(t\right),\\ {\dot{x}}_{i}\left(t\right)=x_{i+1}^{p_{i}}\left(t\right)+\phi_{i}\left({\bar{x}}_{i}\left(t\right)\right)+d_{i}\left(t\right),\;i=2,\ldots ,n-1,\\ {\dot{x}}_{n}\left(t\right)=u^{p_{n}}\left(t\right)+\phi_{n}\left({\bar{x}}_{n}\left(t\right)\right)+d_{n}\left(t\right),\\ y\left(t\right)=x_{1}\left(t\right), \end{array}\right.$$

where $x_{i}\left(t\right)\in R,\;i=1,\ldots ,n$ denotes the system state, and $u\left(t\right)\in R,\;d_{i}\left(t\right)\in R$, and y(t)∈R, respectively, represent control input, unknown disturbances, and system output. z,i=1,…,n are some positive odd integers, and $\phi_{i}\left(\cdot \right),\;i=1,\ldots ,n$ are known nonlinear continuous functions. Moreover, we define $y_{d}\left(t\right)$ as the given reference signal and E(t) as the tracking error between y(t) and $y_{d}\left(t\right)$.

In order to solve the anti-disturbance and prescribed performance tracking control problem of system (1), we aimed to develop a novel composite controller that meets the following control objectives for system (1):

1. The tracking error E(t) converges to zero and achieves the prescribed performance in both transient state and steady state.
2. All states in the closed-loop system are stable.

**Assumption 1.**  *Define $p_{i},\;i=1,\ldots ,n$ as positive odd integers:*

*(i)*  *p is considered as $p=\max\{p_{i}\},\;i=1,\ldots ,n.$*
*(ii)*  *$p_{i}$ satisfies: $\frac{p+1}{p_{i}}\geq p-p_{i+1}+1,\;i=1,\ldots ,n-1$.*

**Assumption 2.**  *The disturbances satisfy the following conditions:*

*(i)*  *$d_{i}\left(t\right)$ and the derivatives of ${\dot{d}}_{i}\left(t\right)$ are bounded, and $d_{i}\left(t\right)$ are nonvanishing.*
*(ii)*  *${\dot{d}}_{i}\left(t\right)\to 0$ as t→∞.*

**Assumption 3.**  *The expected signal $y_{d}\left(t\right)$ and its i-order derivative $y_{d}^{\left(i\right)}\left(t\right)$ are bounded, and they are known.*

**Remark 1.**  *Assumption 1 is utilized to ensure the reasonableness of the adding a power-integrator technology. Assumption 2 is widely used in the field of disturbance estimation for the reason that the derivatives of the disturbances will affect the convergence of error dynamics equation, and this assumption is essential in analyzing the stability of disturbance estimation error. It is worth pointing out that Assumption 3 is a standard assumption for output tracking control of nonlinear systems, and similar assumptions can be found in the literature [14,15,16].*

**Definition 1.**  *A continuous function η(t):[0,b)→[0,∞) is said to belong to class K if it is strictly increasing and η(0)=0. Additionally, it is said to belong to class $K_{\infty }$ if b=∞ and η(s)→∞ as s→∞.*

**Lemma 1**  ([36]). *Consider the following system:*

(2) $$\dot{x}\left(t\right)=f\left(t,x\left(t\right),u\left(t\right)\right),\;x\left(t\right)\in R^{n},u\left(t\right)\in R^{m}.$$

*Let V(t,x) be a continuously differentiable function such that*

$$\begin{array}{cc} \; & \pi_{1}\left(\|x\left(t\right)\|\right)\leq V\left(t,x\right)\leq \pi_{2}\left(\|x\left(t\right)\|\right),\\ \; & \frac{\partial V}{\partial t}+\frac{\partial V}{\partial x}f\left(t,x\left(t\right),u\left(t\right)\right)\leq -\pi_{3}\left(x\left(t\right)\right),\;\forall \|x\left(t\right)\|\geq \pi_{4}\left(\|u\left(t\right)\|\right)>0, \end{array}$$

*where $\pi_{1}\left(\cdot \right)$ and $\pi_{2}\left(\cdot \right)$ are class $K_{\infty }$ functions, $\pi_{3}\left(\cdot \right)$ is a continuous positive definite function, and $\pi_{4}\left(\cdot \right)$ is a class K function. Then, system (2) is input-to-state stable (ISS).*

**Lemma 2**  ([36]). *Consider system (2). If it is globally input-to-state stable, $\lim_{t\to \infty }u\left(t\right)=0,$ then the state of system (2) will asymptotically converge to zero; that is, $\lim_{t\to \infty }x\left(t\right)=0.$*

To complete this section, we give other existing inequalities as lemmas, which the main method of the modified adding a power-integrator technology is based on, and it will be utilized to deal with the error system.

**Lemma 3**  ([37]). *For any real valued function x, y and any positive odd integer q≥1, the inequality is as follows: $|x^{q}-y^{q}|\leq q|x-y|\left(x^{q-1}+y^{q-1}\right).$*

**Lemma 4**  ([37]). *For any designed constant q≥0, the following inequality hold:*

$$|x+y{|}^{q}\leq \max\left\{2^{q-1},1\right\}\left(|x{|}^{q}+|y{|}^{q}\right).$$

*In this paper, because of the uncertainty of $q=p_{i}-1$, the relation between q and* *1 requires further discussion. Thus, to simplify the proof later, the situation that includes both above cases (q<1 and q≥1) is summed up in the following inequality:*

$$|x+y{|}^{q}\leq 2^{q}\left(|x{|}^{q}+|y{|}^{q}\right).$$

**Lemma 5**  ([38]). *For any positive real numbers m and n and any real number ε>0, there are always any real variables x and y and a function a(x,y) such that the following inequality with two forms holds:*

$$|x{|}^{m}|y{|}^{n}\leq \frac{m}{m+n}\varepsilon |x{|}^{m+n}+\frac{m}{m+n}\varepsilon^{-\frac{m}{n}}|y{|}^{m+n},$$

$$|a\left(x,y\right)x^{m}y^{n}|\leq c\left(x,y\right)|x{|}^{m+n}+\frac{m}{m+n}{\left(\frac{m}{\left(m+n)c(x,y\right)}\right)}^{-\frac{m}{n}}|a\left(x,y\right){|}^{\frac{m+n}{n}}|y{|}^{m+n},$$

*where c(x,y)>0.*

### 2.2. Prescribed Performance

In order to guarantee the transient and steady-state performance of tracking error $E\left(t\right)=y\left(t\right)-y_{d}\left(t\right)$ simultaneously, a positive decreasing smooth function $\nu \left(t\right)\;:\;R^{+}\to R^{+}$ was chosen as the prescribed performance function (PPF) with $\lim_{t\to \infty }\nu \left(t\right)=\nu_{\infty }>0.$ In this research, ν(t) was chosen as

$$\nu \left(t\right)=\left(\nu_{0}-\nu_{\infty }\right)e^{-\rho (t)t}+\nu_{\infty },$$

$$\rho \left(t\right)=\frac{\rho_{\infty }\left[\tanh(\varepsilon_{0}\left(t-t_{0}\right))+1\right]}{2},$$

where $\rho_{\infty },\;\varepsilon_{0},\;t_{0}$ and $\nu_{0}>\nu_{\infty }$ are positive parameters which will be designed according to practical requirements.

Utilizing the similar idea from research [29], the prescribed performance can be guaranteed by achieving

(3) $$-\underline{\delta }\nu \left(t\right)<E\left(t\right)<\bar{\delta }\nu \left(t\right),\;\forall t>0,$$

where $\underline{\delta }>0$ and $\bar{\delta }>0$ are constants. Additionally, it must be pointed out that $\nu_{0},\;\underline{\delta }$ and $\bar{\delta }$ should be chosen such that $-\underline{\delta }\nu \left(0\right)<E\left(0\right)<\bar{\delta }\nu \left(0\right).$

**Remark 2.**  *The principle of PPC is to transform the tracking error constrained by the performance function into an unconstrained error that is better handled. Reference [29] stated that the prescribed performance is guaranteed when the tracking error converges to an arbitrarily small set of residuals and the convergence rate and maximum overshoot are less than prescribed values. Therefore, to solve the control problem with prescribed performance (3), a smooth and strictly increasing function T(χ(t)) of the transformed error χ(t)∈R is defined which satisfies*

*(i)* 
$-\underline{\delta }<T\left(\chi \left(t\right)\right)<\bar{\delta },\;\forall \chi \left(t\right)\in L_{\infty },$

*(ii)* 
$\lim_{\chi (t)\to +\infty }T\left(\chi \left(t\right)\right)=\bar{\delta },\lim_{\chi (t)\to -\infty }T\left(\chi \left(t\right)\right)=\underline{\delta }.$

For the properties of T(χ(t)), condition (3) equals

(4) E(t)=ν(t)T(χ(t)).

As T(χ(t)) is strictly monotonically increasing and $\nu \left(t\right)\geq \nu_{\infty }>0,$ the inverse function can be written as

(5) $$\chi \left(t\right)=T^{-1}\left(\frac{E(t)}{\nu (t)}\right).$$

From the above analysis, it can be observed that if χ(t) is bounded, then the prescribed performance (3) can be guaranteed. To facilitate the control design to stabilize χ(t) in (4), the transformed function T(χ(t)) can be chosen as

$$T\left(\chi \left(t\right)\right)=\frac{\bar{\delta }e^{\chi (t)}-\underline{\delta }e^{-\chi (t)}}{e^{\chi (t)}+e^{-\chi (t)}};$$

moreover, from (5), the transformed error χ(t) can be deduced as

$$\chi \left(t\right)=T^{-1}\left(\frac{E(t)}{\nu (t)}\right)=\frac{1}{2}\ln\frac{T\left(\chi \left(t\right)\right)+\underline{\delta }}{\bar{\delta }-T\left(\chi \left(t\right)\right)}.$$

Therefore, based on (1), (4), and (5), the derivation of transformed error χ(t) is derived as

(6) $$\begin{array}{cc} \dot{\chi }\left(t\right)= & \frac{1}{2}{\left(\ln\frac{T\left(\chi \left(t\right)\right)+\underline{\delta }}{\bar{\delta }-T\left(\chi \left(t\right)\right)}\right)}^{\prime }\\ = & \frac{1}{2}{\left(\ln\left(T\left(\chi \left(t\right)\right)+\underline{\delta }\right)-\ln\left(\bar{\delta }-T\left(\chi \left(t\right)\right)\right)\right)}^{\prime }\\ = & \frac{1}{2}\left(\frac{1}{T\left(\chi \left(t\right)\right)+\underline{\delta }}-\frac{1}{\bar{\delta }-T\left(\chi \left(t\right)\right)}\right)\dot{T}\left(\chi \left(t\right)\right)\\ = & \frac{1}{2}\left(\frac{1}{T\left(\chi \left(t\right)\right)+\underline{\delta }}-\frac{1}{\bar{\delta }-T\left(\chi \left(t\right)\right)}\right)\frac{\dot{E}\left(t\right)\nu \left(t\right)-E\left(t\right)\dot{\nu }\left(t\right)}{\nu^{2}\left(t\right)}\\ = & \Gamma \left(x_{2}^{p_{1}}\left(t\right)+\phi_{1}\left({\bar{x}}_{1}\left(t\right)\right)+d_{1}\left(t\right)-\dot{y_{d}\left(t\right)}-\Upsilon \right), \end{array}$$

where $\Gamma =\frac{1}{2\nu (t)}\left(\frac{1}{\frac{E(t)}{\nu (t)}+\underline{\delta }}-\frac{1}{\bar{\delta }-\frac{E(t)}{\nu (t)}}\right)>0$ and $\Upsilon =\frac{E\dot{\nu }\left(t\right)}{\nu (t)}$.

**Remark 3.**  *It is noted that $\bar{\delta }\nu \left(0\right)$ specifies the upper bound of the maximum overshoot, and $-\underline{\delta }\nu \left(0\right)$ represents the lower one; the decreasing rate of ν(t) embodies a lower bound on the needed speed of convergence of E(t), which is drawn to ρ(t). Furthermore, on behalf of the maximum allowable size of the tracking error at the steady state, the positive parameter $\nu_{\infty }=\lim_{t\to \infty }\nu \left(t\right)$ can be selected to be arbitrarily small to promote the tracking accuracy.*

### 2.3. Disturbance Observer

In system (1), the disturbance $d_{i}\left(t\right)$ is unknown. For estimating ${\hat{d}}_{i}\left(t\right)$, the following nonlinear DOB is designed as

(7) $$\begin{array}{cc} \; & {\hat{d}}_{i}\left(t\right)=\lambda_{i}\left(x_{i}\left(t\right)-p_{i}\left(t\right)\right),\\ \; & {\dot{p}}_{i}\left(t\right)=x_{i+1}^{p_{i}}\left(t\right)+\phi_{i}\left({\bar{x}}_{i}\left(t\right)\right)+{\hat{d}}_{i}\left(t\right), \end{array}$$

where $x_{n+1}\left(t\right)=u\left(t\right)$, $p_{i}\left(t\right)$ represents the internal states of the DOB, and $\lambda_{i}>0$.

From (7), we know

(8) $$\begin{array}{c} {\dot{\hat{d}}}_{i}\left(t\right)=\lambda_{i}\left(x_{i}\left(t\right)-p_{i}\left(t\right)\right)=\lambda_{i}\left(d_{i}\left(t\right)-{\hat{d}}_{i}\left(t\right)\right). \end{array}$$

Let $e_{i}\left(t\right)=d_{i}\left(t\right)-{\hat{d}}_{i}\left(t\right)$, based on (1), (7) and (8). Then, the disturbance estimation error system can be described as

(9) $$\begin{array}{c} {\dot{e}}_{i}\left(t\right)={\dot{d}}_{i}\left(t\right)-{\dot{\hat{d}}}_{i}\left(t\right)=-\lambda_{i}e_{i}\left(t\right)+{\dot{d}}_{i}\left(t\right). \end{array}$$

**Remark 4.**  *In the actual control system, it is necessary to design a controller with robustness for avoiding the influences of model uncertainty, parameter perturbation, external disturbances, and other factors. The DOB-based controller can effectively eliminate the influence caused by the above factors. In addition, the composite controller with DOB can be divided into two parts, inner ring and outer ring, which is convenient for design and implementation. Concretely speaking, the inner ring can improve the robustness of the system, and the outer one can be flexibly designed to achieve control objection. Additionally, through the compensation of equivalent disturbance by DOB, the system can present the nominal performance, thereby facilitating the design of the outer-ring of controller.*

## 3. Main Results

This section can be divided into two parts. First, a composite controller is recursively designed by means of the back-stepping method and the nonlinear disturbance observer constructed above. Secondly, the main results of this paper are derived from two theorems with strict proofs.

### 3.1. Composite Controller

(10) $$\left\{\begin{array}{c} {\dot{\chi }}_{1}=\Gamma \left(x_{2}^{p_{1}}\left(t\right)+\phi_{1}\left({\bar{x}}_{1}\left(t\right)\right)+d_{1}\left(t\right)-\dot{y_{d}}\left(t\right)-\Upsilon \right),\\ {\dot{x}}_{i}\left(t\right)=x_{i+1}^{p_{i}}\left(t\right)+\phi_{i}\left({\bar{x}}_{i}\left(t\right)\right)+d_{i}\left(t\right),\;\;i=2,\ldots ,n-1,\\ {\dot{x}}_{n}\left(t\right)=u^{p_{n}}\left(t\right)+\phi_{n}\left({\bar{x}}_{n}\left(t\right)\right)+d_{n}\left(t\right).\\ y\left(t\right)=x_{1}\left(t\right). \end{array}\right.$$

Then, as the preparatory design of the whole composite controller, we introduce

(11) $$\left\{\begin{array}{c} s_{1}\left(t\right)=\chi \left(t\right)-\frac{1}{2}\ln\frac{\underline{\delta }}{\bar{\delta }},\\ s_{i}\left(t\right)=x_{i}\left(t\right)-\alpha_{i}\left(t\right),\;i=2,\ldots ,n,\\ x_{n+1}\left(t\right)=\alpha_{n+1}\left(t\right)=u\left(t\right), \end{array}\right.$$

where $\alpha_{i}\left(t\right)$ denotes the virtual control input to be determined for the *i*th subsystem.

For simplifying the expression of functions, a function f(x(t)) can be rewritten as f(x) or *f* in the following analysis. The design procedures of back-stepping composite controller are given as follows:

**STEP 1.** Consider the first subsystem as

(12) $${\dot{s}}_{1}\left(t\right)=\Gamma \left(x_{2}^{p_{1}}\left(t\right)+\phi_{1}\left({\bar{x}}_{1}\left(t\right)\right)+d_{1}\left(t\right)-\dot{y_{d}}\left(t\right)-\Upsilon \right).$$

Choose a Lyapunov function as

(13) $$V_{1}=\frac{s_{1}^{p-p_{1}+2}\left(t\right)}{p-p_{1}+2}+\frac{e_{1}^{p-p_{1}+2}\left(t\right)}{p-p_{1}+2}.$$

According to (9) and (12), the time derivative of $V_{1}$ yields

(14) $$\begin{array}{c} \begin{array}{cc} {\dot{V}}_{1}= & s_{1}^{p-p_{1}+1}\dot{s_{1}}+e_{1}^{p-p_{1}+1}\dot{e_{1}}\\ = & s_{1}^{p-p_{1}+1}\left[\Gamma \left(x_{2}^{p_{1}}+\phi_{1}\left({\bar{x}}_{1}\right)+d_{1}-\dot{y_{d}}-\Upsilon \right)\right]-\lambda_{1}e_{1}^{p-p_{1}+2}+e_{1}^{p-p_{1}+1}{\dot{d}}_{1}\\ = & s_{1}^{p-p_{1}+1}\Gamma \left(\alpha_{2}^{p_{1}}+\phi_{1}\left({\bar{x}}_{1}\right)+{\hat{d}}_{1}-\dot{y_{d}}-\Upsilon \right)-\lambda_{1}e_{1}^{p-p_{1}+2}+e_{1}^{p-p_{1}+1}{\dot{d}}_{1}\\ \; & +s_{1}^{p-p_{1}+1}\Gamma e_{1}+s_{1}^{p-p_{1}+1}\Gamma \left(x_{2}^{p_{1}}-\alpha_{2}^{p_{1}}\right), \end{array} \end{array}$$

through the help of Lemmas 3 and 4, and (11), one gets

(15) $$\begin{array}{cc} \; & \left|s_{1}^{p-p_{1}+1}\Gamma \left(x_{2}^{p_{1}}-\alpha_{2}^{p_{1}}\right)\right|\\ \leq & \Gamma p_{1}|s_{1}{|}^{p-p_{1}+1}\left|x_{2}-\alpha_{2}\right|\left(x_{2}^{p_{1}-1}+\alpha_{2}^{p_{1}-1}\right)\\ = & \Gamma p_{1}|s_{1}{|}^{p-p_{1}+1}\left|s_{2}\right|\left[{\left(s_{2}+\alpha_{2}\right)}^{p_{1}-1}+\alpha_{2}^{p_{1}-1}\right]\\ \leq & \Gamma p_{1}|s_{1}{|}^{p-p_{1}+1}\left|s_{2}\right|\left[2^{p_{1}-1}\left(|s_{2}{|}^{p_{1}-1}+{\left|\alpha_{2}\right|}^{p_{1}-1}\right)+{\left|\alpha_{2}\right|}^{p_{1}-1}\right]\\ = & 2^{p_{1}-1}\Gamma p_{1}|s_{1}{|}^{p-p_{1}+1}|s_{2}{|}^{p_{1}}+\left(2^{p_{1}-1}+1\right)\Gamma p_{1}|s_{1}{|}^{p-p_{1}+1}|s_{2}\left|\right|\alpha_{2}{|}^{p_{1}-1}, \end{array}$$

and by applying the first inequality of Lemma 5 with $m=p-p_{1}+1,\;n=p_{1},\;\varepsilon =\frac{p+1}{p-p_{1}+1}\frac{1}{p_{1}2^{p_{1}}}$, we obtain

(16) $$\begin{array}{cc} \Gamma p_{1}|s_{1}{|}^{p-p_{1}+1}2^{p_{1}-1}{\left|s_{2}\right|}^{p_{1}}\leq & p_{1}2^{p_{1}-1}\frac{p-p_{1}+1}{p+1}\left(\frac{p+1}{p-p_{1}+1}\frac{1}{p_{1}2^{p_{1}}}\right){\left|s_{1}\right|}^{p+1}\\ \; & +p_{1}2^{p_{1}-1}{\left(\frac{p+1}{p-p_{1}+1}\frac{1}{p_{1}2^{p_{1}}}\right)}^{-\frac{p-p_{1}+1}{p+1}}{\left|\Gamma^{\frac{1}{p_{1}}}s_{2}\right|}^{p+1}\\ \leq & \frac{1}{2}s_{1}^{p+1}+s_{2}^{p+1}\Gamma^{\frac{p+1}{p_{1}}}\beta_{11}, \end{array}$$

where $\beta_{11}=p_{1}2^{p_{1}-1}{\left(\frac{p+1}{p-p_{1}+1}\frac{1}{p_{1}2^{p_{1}}}\right)}^{-\frac{p-p_{1}+1}{p+1}}$.

Using the same m,n, let $\varepsilon =\frac{p+1}{p-p_{1}+1}\frac{1}{p_{1}\left(2^{p_{1}}+2\right)}$ of the first inequality of Lemma 5. This yields

(17) $$\begin{array}{cc} \; & \Gamma p_{1}|s_{1}{|}^{p-p_{1}+1}\left(2^{p_{1}-1}+1\right)|s_{2}\left|\right|\alpha_{2}{|}^{p_{1}-1}\\ \leq & p_{1}\left(2^{p_{1}-1}+1\right)\frac{p-p_{1}+1}{p+1}\left(\frac{p+1}{p-p_{1}+1}\frac{1}{p_{1}\left(2^{p_{1}}+2\right)}\right){\left|s_{1}\right|}^{p+1}\\ \; & +p_{1}\left(2^{p_{1}-1}+1\right)\frac{p_{1}}{p+1}{\left(\frac{p+1}{p-p_{1}+1}\frac{1}{p_{1}\left(2^{p_{1}}+2\right)}\right)}^{-\frac{p-p_{1}+1}{p+1}}{\left|\Gamma^{\frac{1}{p_{1}}}s_{2}^{\frac{1}{p_{1}}}\alpha_{2}^{\frac{p_{1}-1}{p_{1}}}\right|}^{p+1}\\ \leq & \frac{1}{2}s_{1}^{p+1}+s_{2}^{\frac{p+1}{p_{1}}}\Gamma^{\frac{p+1}{p_{1}}}\beta_{12}, \end{array}$$

where $\beta_{12}=\frac{\left(2^{p_{1}-1}+1\right)p_{1}^{2}}{p+1}{\left(\frac{p+1}{p-p_{1}+1}\frac{1}{p_{1}\left(2^{p_{1}}+2\right)}\right)}^{-\frac{p-p_{1}+1}{p_{1}}}\alpha_{2}^{\frac{\left(p+1\right)\left(p_{1}-1\right)}{p_{1}}}$.

Meanwhile, with the help of the second inequality of Lemma 5, let m=1 and $n=p-p_{1}+1,\;a\left(x,y\right)=\Gamma$. We get

(18) $$\begin{array}{c} |e_{1}\Gamma s_{1}^{p-p_{1}+1}|\leq c_{1}|e_{1}{|}^{p-p_{1}+2}+a_{1}\Gamma^{\frac{p-p_{1}+2}{p-p_{1}+1}}{\left|s_{1}\right|}^{p-p_{1}+2}, \end{array}$$

where $a_{1}=\frac{1}{p-p_{1}+2}{\left(\frac{1}{\left(p-p_{1}+2\right)c_{1}}\right)}^{\frac{1}{p-p_{1}+1}},\;c_{1}>0$.

Now, we select

(19) $$\begin{array}{c} \alpha_{2}={\left(-\frac{k_{1}s_{1}+s_{1}^{p_{1}}}{\Gamma }-\phi \left({\bar{x}}_{1}\right)-\hat{d_{1}}+y_{d}+\Upsilon -a_{1}\Gamma^{\frac{p-p_{1}+2}{p-p_{1}+1}}s_{1}\right)}^{\frac{1}{p_{1}}}, \end{array}$$

where $k_{1}>0.$

By substituting (15)–(18) and the control law (19) into (14), the latter is rewritten as

(20) $$\begin{array}{c} {\dot{V}}_{1}\leq -k_{1}s_{1}^{p-p_{1}+2}+s_{2}^{p+1}\Gamma^{\frac{p+1}{p_{1}}}\beta_{11}+s_{2}^{\frac{p+1}{p_{1}}}\Gamma^{\frac{p+1}{p_{1}}}\beta_{12}-\left(\lambda_{1}-c_{1}\right)e_{1}^{p-p_{1}+2}+e_{1}^{p-p_{1}+1}{\dot{d}}_{1}. \end{array}$$

**STEP 2.** Based on $s_{2}=x_{2}-\alpha_{2}$, (10) and (19), we have

(21) $$\begin{array}{cc} {\dot{s}}_{2}= & {\dot{x}}_{2}-{\dot{\alpha }}_{2}\\ = & x_{3}^{p_{2}}+\phi_{2}\left({\bar{x}}_{2}\right)+d_{2}-\sum_{j=0}^{1}\frac{\partial \alpha_{2}}{\partial \nu {\left(t\right)}^{\left(j\right)}}\nu {\left(t\right)}^{\left(j+1\right)}-\sum_{j=0}^{1}\frac{\partial \alpha_{2}}{\partial y_{d}^{\left(j\right)}}y_{d}^{\left(j+1\right)}\\ \; & -\frac{\partial \alpha_{2}}{\partial x_{1}}\left(x_{2}^{p_{1}}+\phi_{1}\left({\bar{x}}_{1}\right)+d_{1}\right)-\frac{\partial \alpha_{2}}{\partial {\hat{d}}_{1}}\lambda_{1}e_{1}. \end{array}$$

Choose the following Lyapunov function

(22) $$\begin{array}{c} V_{2}=V_{1}+\frac{s_{2}^{p-p_{2}+2}}{p-p_{2}+2}+\frac{e_{2}^{p-p_{2}+2}}{p-p_{2}+2}. \end{array}$$

Combine (9), (20) and (21). The derivative of $V_{2}$ is depicted by

(23) $$\begin{array}{cc} {\dot{V}}_{2}= & {\dot{V}}_{1}+s_{2}^{p-p_{2}+1}\dot{s_{2}}+e_{2}^{p-p_{2}+1}\dot{e_{2}}\\ \leq & -k_{1}s_{1}^{p-p_{1}+2}+s_{2}^{p+1}\Gamma^{\frac{p+1}{p_{1}}}\beta_{11}+s_{2}^{\frac{p+1}{p_{1}}}\Gamma^{\frac{p+1}{p_{1}}}\beta_{12}\\ \; & +s_{2}^{p-p_{2}+1}(x_{3}^{p_{2}}+\phi_{2}\left({\bar{x}}_{2}\right)+d_{2}-\sum_{j=0}^{1}\frac{\partial \alpha_{2}}{\partial \nu {\left(t\right)}^{\left(j\right)}}\nu {\left(t\right)}^{\left(j+1\right)}-\sum_{j=0}^{1}\frac{\partial \alpha_{2}}{\partial y_{d}^{\left(j\right)}}y_{d}^{\left(j+1\right)}\\ \; & -\frac{\partial \alpha_{2}}{\partial x_{1}}\left(x_{2}^{p_{1}}+\phi_{1}\left({\bar{x}}_{1}\right)+d_{1}\right)-\frac{\partial \alpha_{2}}{\partial {\hat{d}}_{1}}\lambda_{1}e_{1})\\ \; & -\left(\lambda_{1}-c_{1}\right)e_{1}^{p-p_{1}+2}+e_{1}^{p-p_{1}+1}{\dot{d}}_{1}-\lambda_{2}e_{2}^{p-p_{2}+2}+e_{2}^{p-p_{2}+1}{\dot{d}}_{2}\\ \leq & -k_{1}s_{1}^{p-p_{1}+2}-s_{2}^{p-p_{2}+1}\frac{\partial \alpha_{2}}{\partial x_{1}}e_{1}+s_{2}^{p-p_{2}+1}\left(x_{3}^{p_{2}}-\alpha_{3}^{p_{2}}\right)\\ \; & +s_{2}^{p-p_{2}+1}[\alpha_{3}^{p_{2}}+s_{2}^{p_{2}}\Gamma^{\frac{p+1}{p_{1}}}\beta_{11}+s_{2}^{{\bar{P}}_{2}}\Gamma^{\frac{p+1}{p_{1}}}\beta_{12}+\phi_{2}{\bar{x}}_{2}+e_{2}+{\hat{d}}_{2}\\ \; & -\sum_{j=0}^{1}\frac{\partial \alpha_{2}}{\partial \nu {\left(t\right)}^{\left(j\right)}}\nu {\left(t\right)}^{\left(j+1\right)}-\sum_{j=0}^{1}\frac{\partial \alpha_{2}}{\partial y_{d}^{\left(j\right)}}y_{d}^{\left(j+1\right)}-\frac{\partial \alpha_{2}}{\partial x_{1}}\left(x_{2}^{p_{1}}+\phi_{1}\left({\bar{x}}_{1}\right)+{\hat{d}}_{1}\right)-\frac{\partial \alpha_{2}}{\partial {\hat{d}}_{1}}\lambda_{1}e_{1}]\\ \; & -\left(\lambda_{1}-c_{1}\right)e_{1}^{p-p_{1}+2}+e_{1}^{p-p_{1}+1}{\dot{d}}_{1}-\lambda_{2}e_{2}^{p-p_{1}+2}+e_{2}^{p-p_{1}+1}{\dot{d}}_{2}, \end{array}$$

where ${\bar{p}}_{2}=\frac{p+1}{p_{1}}-\left(p-p_{2}+1\right)$ is a non-negative constant under the second condition of Assumption 1.

Currently, $\alpha_{3}$ is designed as

(24) $$\begin{array}{cc} \alpha_{3}= & [-s_{2}^{p_{2}}-\left(a_{2}+{\hat{a}}_{2}+k_{2}\right)s_{2}-s_{2}^{p_{2}}\Gamma^{\frac{p+1}{p_{1}}}\beta_{11}-s_{2}^{{\bar{P}}_{2}}\Gamma^{\frac{p+1}{p_{1}}}\beta_{12}-\phi_{2}{\bar{x}}_{2}-{\hat{d}}_{2}\\ \; & +\sum_{j=0}^{1}\frac{\partial \alpha_{2}}{\partial \nu {\left(t\right)}^{\left(j\right)}}\nu {\left(t\right)}^{\left(j+1\right)}+\sum_{j=0}^{1}\frac{\partial \alpha_{2}}{\partial y_{d}^{\left(j\right)}}y_{d}^{\left(j+1\right)}+\frac{\partial \alpha_{2}}{\partial x_{1}}\left(x_{2}^{p_{1}}+\phi_{1}\left({\bar{x}}_{1}\right)+{\hat{d}}_{1}\right)+\frac{\partial \alpha_{2}}{\partial {\hat{d}}_{1}}\lambda_{1}e_{1}{]}^{\frac{1}{p_{2}}}, \end{array}$$

where $k_{2}>0$, $a_{2}$, and ${\hat{a}}_{2}$ are designed next.

Meanwhile, by applying Lemma 5, it follows that

(25) $$\begin{array}{c} \begin{array}{cc} \; & |e_{2}s_{2}^{p-p_{2}+1}|\leq c_{2}|e_{2}{|}^{p-p_{2}+2}+a_{2}{\left|s_{2}\right|}^{p-p_{2}+2},\\ \; & -s_{2}^{p-p_{2}+1}\left(\frac{\partial \alpha_{2}}{\partial x_{1}}+\frac{\partial \alpha_{2}}{\partial {\hat{d}}_{1}}\lambda_{1}\right)e_{1}\leq c_{1}e_{1}^{p-p_{2}+2}+{\hat{a}}_{2}s_{2}^{p-p_{2}+2}, \end{array} \end{array}$$

where $c_{2}>0,a_{2}=\frac{p-p_{2}+1}{p-p_{2}+2}{\left(\frac{1}{\left(p-p_{2}+2\right)c_{2}}\right)}^{\frac{1}{p-p_{2}+1}},\;$${\hat{a}}_{2}=\frac{p-p_{2}+1}{p-p_{2}+2}{\left(\frac{1}{\left(p-p_{2}+2\right)c_{1}}\right)}^{\frac{1}{p-p_{2}+1}}{\left(\frac{\partial \alpha_{2}}{\partial x_{1}}+\frac{\partial \alpha_{2}}{\partial {\hat{d}}_{1}}\lambda_{1}\right)}^{p-p_{2}+2}$.

A similar argument for (15)–(17) in Step 1 leads to

(26) $$\begin{array}{cc} s_{2}^{p-p_{2}+1}\left(x_{3}^{p_{2}}-\alpha_{3}^{p_{2}}\right)\leq & 2^{p_{2}-1}p_{2}|s_{2}{|}^{p-p_{2}+1}|s_{3}{|}^{p_{2}}+\left(2^{p_{2}-1}+1\right)p_{2}|s_{2}{|}^{p-p_{2}+1}|s_{3}\left|\right|\alpha_{3}{|}^{p_{2}-1}\\ \leq & s_{2}^{p+1}+s_{3}^{p+1}\beta_{21}+s_{3}^{\frac{p+1}{p_{2}}}\beta_{22}, \end{array}$$

where $\beta_{21}=p_{2}2^{p_{2}-1}\frac{p_{2}}{p+1}{\left(\frac{p+1}{p-p_{2}+1}\frac{1}{p_{2}2^{p_{2}}}\right)}^{-\frac{p-p_{2}+1}{p_{2}}}$, and $\;\beta_{22}=\frac{\left(2^{p_{2}-1}+1\right)p_{2}^{2}}{p+1}$${\left(\frac{p+1}{p-p_{2}+1}\frac{1}{p_{2}\left(2^{p_{2}}+2\right)}\right)}^{-\frac{p-p_{2}+1}{p_{2}}}\alpha_{3}^{\frac{\left(p+1\right)\left(p_{2}-1\right)}{p_{2}}}$.

By substituting (25) and (26) and the control law (24) into (23), it is rewritten as

(27) $$\begin{array}{c} \begin{array}{cc} {\dot{V}}_{2}\leq & \sum_{j=1}^{2}k_{j}s_{j}^{p-p_{j}+2}+s_{3}^{p+1}\beta_{21}+s_{3}^{\frac{p+1}{p_{2}}}\beta_{22}\\ \; & +\sum_{j=1}^{2}e_{j}^{p-p_{j}+1}{\dot{d}}_{j}-\left(\lambda_{2}-c_{2}\right)e_{2}^{p-p_{2}+2}-\left(\lambda_{1}-2c_{1}\right)e_{1}^{p-p_{1}+2}. \end{array} \end{array}$$

**STEP 3.** Similarly to Step 2, consider $s_{3}=x_{3}-\alpha_{3}$ and (24). We have

(28) $$\begin{array}{cc} {\dot{s}}_{3}= & {\dot{x}}_{3}-{\dot{\alpha }}_{3}\\ = & x_{4}^{p_{3}}+\phi_{3}\left({\bar{x}}_{3}\right)+d_{3}-\sum_{j=0}^{2}\frac{\partial \alpha_{3}}{\partial \nu {\left(t\right)}^{\left(j\right)}}\nu {\left(t\right)}^{\left(j+1\right)}-\sum_{j=0}^{2}\frac{\partial \alpha_{3}}{\partial y_{d}^{\left(j\right)}}y_{d}^{\left(j+1\right)}\\ \; & -\sum_{j=1}^{2}\frac{\partial \alpha_{3}}{\partial x_{j}}\left(x_{j+1}^{p_{j}}+\phi_{j}\left({\bar{x}}_{j}\right)+d_{j}\right)-\sum_{j=1}^{2}\frac{\partial \alpha_{3}}{\partial {\hat{d}}_{j}}\lambda_{j}e_{j}. \end{array}$$

Choose the following Lyapunov function:

$$\begin{array}{c} V_{3}=V_{2}+\frac{s_{3}^{p-p_{3}+2}}{p-p_{3}+2}+\frac{e_{3}^{p-p_{3}+2}}{p-p_{3}+2}. \end{array}$$

By combining (9), (27), and (28), the derivative of $V_{3}$ is concluded as

(29) $$\begin{array}{cc} {\dot{V}}_{3}= & {\dot{V}}_{2}+s_{3}^{p-p_{j}+1}\dot{s_{3}}+e_{3}^{p-p_{3}+1}\dot{e_{3}}\\ \leq & -\sum_{j=1}^{2}k_{j}s_{j}^{p-p_{j}+2}+s_{3}^{p+1}\beta_{21}+s_{3}^{\frac{p+1}{p_{2}}}\beta_{22}+\sum_{j=1}^{3}e_{j}^{p-p_{i}+1}{\dot{d}}_{j}\\ \; & +s_{3}^{p-p_{3}+1}(x_{4}^{p_{3}}+\phi_{3}\left({\bar{x}}_{3}\right)+d_{3}-\sum_{j=0}^{2}\frac{\partial \alpha_{3}}{\partial \nu {\left(t\right)}^{\left(j\right)}}\nu {\left(t\right)}^{\left(j+1\right)}-\sum_{j=0}^{2}\frac{\partial \alpha_{3}}{\partial y_{d}^{\left(j\right)}}y_{d}^{\left(j+1\right)}\\ \; & -\sum_{j=1}^{2}\frac{\partial \alpha_{3}}{\partial x_{j}}\left(x_{j+1}^{p_{j}}+\phi_{j}\left({\bar{x}}_{j}\right)+d_{j}\right)-\sum_{j=1}^{2}\frac{\partial \alpha_{3}}{\partial {\hat{d}}_{j}}\lambda_{j}e_{j})\\ \; & -\lambda_{3}e_{3}^{p-p_{3}+2}-\left(\lambda_{1}-2c_{1}\right)e_{1}^{p-p_{1}+2}-\left(\lambda_{2}-c_{1}\right)e_{2}^{p-p_{2}+2}\\ \leq & -\sum_{j=1}^{2}k_{j}s_{j}^{p-p_{j}+2}-s_{3}^{p-p_{3}+1}\sum_{j=1}^{2}\frac{\partial \alpha_{3}}{\partial x_{j}}e_{j}+s_{3}^{p-p_{3}+1}\left(x_{4}^{p_{3}}-\alpha_{4}^{p_{3}}\right)\\ \; & +s_{3}^{p-p_{3}+1}[\alpha_{4}^{p_{3}}+s_{3}^{p_{3}}\beta_{21}+s_{3}^{{\bar{p}}_{3}}\beta_{22}+\phi_{3}{\bar{x}}_{3}+e_{3}+{\hat{d}}_{3}-\sum_{j=0}^{2}\frac{\partial \alpha_{3}}{\partial \nu {\left(t\right)}^{\left(j\right)}}\nu {\left(t\right)}^{\left(j+1\right)}\\ \; & -\sum_{j=0}^{2}\frac{\partial \alpha_{3}}{\partial y_{d}^{\left(j\right)}}y_{d}^{\left(j+1\right)}-\sum_{j=1}^{2}\frac{\partial \alpha_{3}}{\partial x_{j}}\left(x_{j+1}^{p_{j}}+\phi_{j}\left({\bar{x}}_{j}\right)+{\hat{d}}_{j}\right)-\sum_{j=1}^{2}\frac{\partial \alpha_{3}}{\partial {\hat{d}}_{j}}\lambda_{j}e_{j}]\\ \; & +\sum_{j=1}^{3}e_{j}^{p-p_{j}+1}{\dot{d}}_{j}-\lambda_{3}e_{3}^{p-p_{3}+2}-\left(\lambda_{1}-2c_{1}\right)e_{1}^{p-p_{1}+2}-\left(\lambda_{2}-c_{2}\right)e_{2}^{p-p_{2}+2}, \end{array}$$

where ${\bar{p}}_{3}=\frac{p+1}{p_{3}-1}-\left(p-p_{3}+1\right)$ is a non-negative constant under the second condition of Assumption 1.

Currently, $\alpha_{4}$ is designed as

(30) $$\begin{array}{cc} \; & \alpha_{4}\\ = & [-s_{3}^{p_{3}}-\left(a_{3}+{\hat{a}}_{3}+k_{3}\right)s_{3}-s_{3}^{p_{3}}\beta_{21}-s_{3}^{{\bar{p}}_{3}}\beta_{22}-\phi_{3}{\bar{x}}_{3}-{\hat{d}}_{3}+\sum_{j=0}^{2}\frac{\partial \alpha_{3}}{\partial \nu {\left(t\right)}^{\left(j\right)}}\nu {\left(t\right)}^{\left(j+1\right)}\\ \; & +\sum_{j=0}^{2}\frac{\partial \alpha_{3}}{\partial y_{d}^{\left(j\right)}}y_{d}^{\left(j+1\right)}+\sum_{j=1}^{2}\frac{\partial \alpha_{3}}{\partial x_{j}}\left(x_{j+1}^{p_{j}}+\phi_{j}\left({\bar{x}}_{j}\right)+{\hat{d}}_{j}\right)+\sum_{j=1}^{2}\frac{\partial \alpha_{3}}{\partial {\hat{d}}_{j}}\lambda_{j}e_{j}{]}^{\frac{1}{p_{3}}}, \end{array}$$

where $k_{3}>0$, $a_{3}$ and ${\hat{a}}_{3}$ are designed later.

Meanwhile, by applying Lemma 5, it follows that

(31) $$\begin{array}{c} \begin{array}{cc} \; & |e_{3}s_{3}^{p-p_{3}+1}|\leq c_{3}|e_{3}{|}^{p-p_{3}+2}+a_{3}{\left|s_{3}\right|}^{p-p_{3}+2},\\ \; & -s_{3}^{p-p_{3}+1}\sum_{j=1}^{2}\left(\frac{\partial \alpha_{3}}{\partial x_{j}}+\frac{\partial \alpha_{3}}{\partial {\hat{d}}_{j}}\lambda_{j}\right)e_{j}\leq \sum_{j=1}^{2}c_{j}|e_{j}{|}^{p-p_{i}+2}+{\hat{a}}_{3}{\left|s_{3}\right|}^{p-p_{3}+2}, \end{array} \end{array}$$

where

$$\begin{array}{c} \begin{array}{cc} \; & c_{3}>0,\\ \; & a_{3}=\frac{p-p_{3}+1}{p-p_{3}+2}{\left(\frac{1}{\left(p-p_{3}+2\right)c_{3}}\right)}^{\frac{1}{p-p_{3}+1}},\\ \; & {\hat{a}}_{3}=\sum_{j=1}^{2}\frac{p-p_{3}+1}{p-p_{3}+2}{\left(\frac{1}{\left(p-p_{3}+2\right)c_{j}}\right)}^{\frac{1}{p-p_{3}+1}}{\left(\frac{\partial \alpha_{3}}{\partial x_{j}}+\frac{\partial \alpha_{3}}{\partial {\hat{d}}_{j}}\lambda_{j}\right)}^{p-p_{3}+2}. \end{array} \end{array}$$

Similarly, the processing of (26) in Step 2 leads to

(32) $$\begin{array}{cc} s_{3}^{p-p_{3}+1}\left(x_{4}^{p_{3}}-\alpha_{4}^{p_{3}}\right)\leq & 2^{p_{3}-1}p_{3}|s_{3}{|}^{p-p_{3}+1}|s_{4}{|}^{p_{3}}+\left(2^{p_{3}-1}+1\right)p_{3}|s_{3}{|}^{p-p_{3}+1}|s_{4}\left|\right|\alpha_{4}{|}^{p_{3}-1}\\ \leq & s_{3}^{p+1}+s_{4}^{p+1}\beta_{31}+s_{4}^{\frac{p+1}{p_{3}}}\beta_{32}, \end{array}$$

where $\beta_{31}=p_{3}2^{p_{3}-1}\frac{p_{3}}{p+1}{\left(\frac{p+1}{p-P_{3}+1}\frac{1}{p_{3}2^{p_{3}}}\right)}^{-\frac{p-p_{3}+1}{p_{3}}}$, and $\beta_{32}=\frac{\left(2^{p_{3}-1}+1\right)p_{3}^{2}}{p+1}$ ${\left(\frac{p+1}{p-p_{3}+1}\frac{1}{p_{3}\left(2^{p_{3}}+2\right)}\right)}^{-\frac{p-p_{3}+1}{p_{3}}}\alpha_{4}^{\frac{\left(p+1\right)\left(p_{3}-1\right)}{p_{3}}}$.

By substituting (31) and (32) and the control law (30) into (29), it is rewritten as

(33) $$\begin{array}{c} \begin{array}{cc} {\dot{V}}_{3}\leq & -\sum_{j=1}^{3}k_{j}s_{j}^{p-p_{j}+2}+s_{4}^{p+1}\beta_{31}+s_{4}^{\frac{p+1}{p_{3}}}\beta_{32}+\sum_{j=1}^{3}e_{j}^{p-p_{j}+1}{\dot{d}}_{j}\\ \; & -\left(\lambda_{3}-c_{3}\right)e_{3}^{p-p_{3}+2}-\left(\lambda_{1}-3c_{1}\right)e_{1}^{p-p_{1}+2}-\left(\lambda_{2}-2c_{2}\right)e_{2}^{p-p_{2}+2}. \end{array} \end{array}$$

**STEP i.** At Step i−1 with i=4,…,n−1, we assume there exists a continuously differential function $V_{i-1}=\sum_{i=1}^{i-1}\frac{s_{j}^{p-p_{j}+2}}{p-p_{j}+2}+\sum_{i=1}^{i-1}\frac{e_{j}^{p-p_{j}+2}}{p-p_{j}+2}$ such that

(34) $$\begin{array}{c} \begin{array}{cc} {\dot{V}}_{i-1}\leq & -\sum_{j=1}^{i-1}k_{j}s_{j}^{p-p_{j}+2}+s_{i}^{p+1}\beta_{i-1,1}+s_{i}^{\frac{p+1}{p_{i-1}}}\beta_{i-1,2}\\ \; & +\sum_{j=1}^{i-1}e_{j}^{p-p_{j}+1}{\dot{d}}_{j}-\sum_{j=1}^{i-1}\left(\lambda_{j}-\left(i-j\right)c_{j}\right)e_{j}^{p-p_{j}+2}. \end{array} \end{array}$$

It is obvious that when i=4, (34) is (33). In what follows, we will give strict proof that (33) also holds at the *i*th step:

For this purpose,

$$\begin{array}{cc} {\dot{s}}_{i}= & {\dot{x}}_{i}-{\dot{\alpha }}_{i}\\ = & x_{i+1}^{p_{i}}+\phi_{i}\left({\bar{x}}_{i}\right)+d_{i}-\sum_{j=0}^{i-1}\frac{\partial \alpha_{i}}{\partial \nu {\left(t\right)}^{\left(j\right)}}\nu {\left(t\right)}^{\left(j+1\right)}-\sum_{j=0}^{i-1}\frac{\partial \alpha_{i}}{\partial y_{d}^{\left(j\right)}}y_{d}^{\left(j+1\right)}\\ \; & -\sum_{j=1}^{i-1}\frac{\partial \alpha_{i}}{\partial x_{j}}\left(x_{j+1}^{p_{j}}+\phi_{j}\left({\bar{x}}_{j}\right)+d_{j}\right)-\sum_{j=1}^{i-1}\frac{\partial \alpha_{i}}{\partial {\hat{d}}_{j}}\lambda_{j}e_{j}, \end{array}$$

and the Lyapunov function $V_{i}$ can be chosen as follows:

(35) $$\begin{array}{c} V_{i}=V_{i-1}+\frac{s_{i}^{p-p_{i}+2}}{p-p_{i}+2}+\frac{e_{i}^{p-p_{i}+2}}{p-p_{i}+2}. \end{array}$$

Enlightened by (9), (11), and (34), we get the following inequality spontaneously:

(36) $$\begin{array}{c} \begin{array}{cc} {\dot{V}}_{i}\leq & -\sum_{j=1}^{i-1}k_{j}s_{j}^{p-p_{j}+2}-s_{i}^{p-p_{i}+1}\sum_{j=1}^{i-1}\frac{\partial \alpha_{i}}{\partial x_{j}}e_{j}+s_{i}^{p-p_{i}+1}\left(x_{i+1}^{p_{i}}-\alpha_{i+1}^{p_{i}}\right)\\ \; & +s_{i}^{p-p_{i}+1}[\alpha_{i+1}^{p_{i}}+s_{i}^{p_{i}}\beta_{i-1,1}+s_{i}^{{\bar{p}}_{i}}\beta_{i-1,2}+\phi_{i}{\bar{x}}_{i}+e_{i}+{\hat{d}}_{i}-\sum_{j=0}^{i_{1}}\frac{\partial \alpha_{i}}{\partial \nu {\left(t\right)}^{\left(j\right)}}\nu {\left(t\right)}^{\left(j+1\right)}\\ \; & -\sum_{j=0}^{i-1}\frac{\partial \alpha_{i}}{\partial y_{d}^{\left(j\right)}}y_{d}^{\left(j+1\right)}-\sum_{j=1}^{i-1}\frac{\partial \alpha_{i}}{\partial x_{j}}\left(x_{j+1}^{p_{j}}+\phi_{j}\left({\bar{x}}_{j}\right)+{\hat{d}}_{j}\right)-\sum_{j=1}^{i-1}\frac{\partial \alpha_{i}}{\partial {\hat{d}}_{j}}\lambda_{j}e_{j}]\\ \; & +\sum_{j=1}^{i}e_{j}^{p-p_{j}+1}{\dot{d}}_{j}-\sum_{j=1}^{i}\left(\lambda_{j}-\left(i-j\right)c_{j}\right)e_{j}^{p-p_{j}+2}-\lambda_{i}e_{i}^{p+p_{i}+2}, \end{array} \end{array}$$

where ${\bar{p}}_{i}=\frac{p+1}{p_{i}-1}-\left(p-p_{i}+1\right)$ is a non-negative constant under the second condition of Assumption 1.

Currently, $\alpha_{i+1}$ is designed as

(37) $$\begin{array}{cc} \; & \alpha_{i+1}=[-s_{i}^{p_{i}}-\left(a_{i}+{\hat{a}}_{i}+k_{i}\right)s_{i}-s_{i}^{p_{i}}\beta_{i-1,1}-s_{i}^{{\bar{p}}_{i}}\beta_{i-1,2}-\phi_{i}{\bar{x}}_{i}-{\hat{d}}_{i}+\sum_{j=0}^{i-1}\frac{\partial \alpha_{i}}{\partial \nu {\left(t\right)}^{\left(j\right)}}\nu {\left(t\right)}^{\left(j+1\right)}\\ \; & +\sum_{j=0}^{i-1}\frac{\partial \alpha_{i}}{\partial y_{d}^{\left(j\right)}}y_{d}^{\left(j+1\right)}+\sum_{j=1}^{i-1}\frac{\partial \alpha_{i}}{\partial x_{j}}\left(x_{j+1}^{p_{j}}+\phi_{j}\left({\bar{x}}_{j}\right)+{\hat{d}}_{j}\right)+\sum_{j=1}^{i-1}\frac{\partial \alpha_{i}}{\partial {\hat{d}}_{j}}\lambda_{j}e_{j}{]}^{\frac{1}{p_{i}}}, \end{array}$$

and $k_{i}>0$, $a_{i}$, and ${\hat{a}}_{i}$ are designed later.

Now applying Lemma 5 again, it follows that

(38) $$\begin{array}{c} \begin{array}{cc} \; & |e_{i}s_{i}^{p-p_{i}+1}|\leq c_{i}|e_{i}{|}^{p-p_{i}+2}+a_{i}{\left|s_{i}\right|}^{p-p_{i}+2},\\ \; & -s_{i}^{p-p_{i}+1}\sum_{j=1}^{i-1}\left(\frac{\partial \alpha_{i}}{\partial x_{j}}+\frac{\partial \alpha_{i}}{\partial {\hat{d}}_{j}}\lambda_{j}\right)e_{j}\leq \sum_{j=1}^{i-1}c_{j}|e_{j}{|}^{p-p_{i}+2}+{\hat{a}}_{i}{\left|s_{i}\right|}^{p-p_{i}+2}, \end{array} \end{array}$$

where

$$\begin{array}{cc} \; & c_{i}>0,\\ \; & a_{i}=\frac{p-p_{i}+1}{p-p_{i}+2}{\left(\frac{1}{\left(p-p_{i}+2\right)c_{i}}\right)}^{\frac{1}{p-p_{i}+1}},\;\\ \; & {\hat{a}}_{i}=\sum_{j=1}^{i-1}\frac{p-p_{i}+1}{p-p_{i}+2}{\left(\frac{1}{\left(p-p_{i}+2\right)c_{j}}\right)}^{\frac{1}{p-p_{i}+1}}{\left(\frac{\partial \alpha_{i}}{\partial x_{j}}+\frac{\partial \alpha_{i}}{\partial {\hat{d}}_{j}}\lambda_{j}\right)}^{p-p_{i}+2}. \end{array}$$

Furthermore,

(39) $$\begin{array}{cc} s_{i}^{p-p_{i}+1}\left(x_{i+1}^{p_{i}}-\alpha_{i+1}^{p_{i}}\right)\leq & 2^{p_{i}-1}p_{i}|s_{i}{|}^{p-p_{i}+1}|s_{i+1}{|}^{p_{i}}+\left(2^{p_{i}-1}+1\right)p_{i}|s_{i}{|}^{p-p_{i}+1}|s_{i+1}\left|\right|\alpha_{i+1}{|}^{p_{i}-1}\\ \leq & s_{i}^{p+1}+s_{i+1}^{p+1}\beta_{i1}+s_{i+1}^{\frac{p+1}{p_{i}}}\beta_{i2}, \end{array}$$

where $\beta_{i1}=p_{i}2^{p_{i}-1}\frac{p_{i}}{p+1}{\left(\frac{p+1}{p-P_{i}+1}\frac{1}{p_{i}2^{p_{i}}}\right)}^{-\frac{p-p_{i}+1}{p_{i}}}$, and $\beta_{i2}=\frac{\left(2^{p_{i}-1}+1\right)p_{i}^{2}}{p+1}$${\left(\frac{p+1}{p-p_{i}+1}\frac{1}{p_{i}\left(2^{p_{i}}+2\right)}\right)}^{-\frac{p-p_{i}+1}{p_{i}}}\alpha_{i+1}^{\frac{\left(p+1\right)\left(p_{i}-1\right)}{p_{i}}}$.

By substituting (38) and (39) and the control law (37) into (36), it is rewritten as

(40) $$\begin{array}{c} \begin{array}{cc} {\dot{V}}_{i}\leq & -\sum_{j=1}^{i}k_{j}s_{j}^{p-p_{j}+2}+s_{i+1}^{p+1}\beta_{i1}+s_{i+1}^{\frac{p+1}{p_{i}}}\beta_{i2}\\ \; & +\sum_{j=1}^{i}e_{j}^{p-p_{j}+1}{\dot{d}}_{j}-\sum_{j=1}^{i}\left(\lambda_{j}-\left(i-j+1\right)c_{j}\right)e_{j}^{p-p_{j}+2}. \end{array} \end{array}$$

**STEP n.** In particular, the actual controller u(t) that we truly need can be found at the last step. ${\dot{s}}_{n}$ can be expressed as

$$\begin{array}{cc} {\dot{s}}_{n}= & {\dot{x}}_{n}-{\dot{\alpha }}_{n}\\ = & x_{n+1}^{p_{n}}+\phi_{n}\left({\bar{x}}_{n}\right)+d_{n}-\sum_{j=0}^{n-1}\frac{\partial \alpha_{i}}{\partial \nu {\left(t\right)}^{\left(j\right)}}\nu {\left(t\right)}^{\left(j+1\right)}-\sum_{j=0}^{n-1}\frac{\partial \alpha_{i}}{\partial y_{d}^{\left(j\right)}}y_{d}^{\left(j+1\right)}\\ \; & -\sum_{j=1}^{n-1}\frac{\partial \alpha_{i}}{\partial x_{j}}\left(x_{j+1}^{p_{j}}+\phi_{j}\left({\bar{x}}_{j}\right)+d_{j}\right)-\sum_{j=1}^{n-1}\frac{\partial \alpha_{i}}{\partial {\hat{d}}_{j}}\lambda_{j}e_{j}, \end{array}$$

and the Lyapunov function $V_{n}$ also can be chosen as

$$\begin{array}{c} V_{n}=V_{n-1}+\frac{s_{n}^{p-p_{n}+2}}{p-p_{n}+2}+\frac{e_{n}^{p-p_{n}+2}}{p-p_{n}+2}. \end{array}$$

Through the same process above, we select

(41) $$\begin{array}{cc} \; & u\left(t\right)=x_{n+1}=\alpha_{n+1}\\ =\; & [-\left(a_{n}+{\hat{a}}_{n}+k_{n}\right)s_{n}-s_{n}^{p_{n}}\beta_{n-1,1}-s_{n}^{{\bar{p}}_{n}}\beta_{n-1,2}-\phi_{n}{\bar{x}}_{n}-{\hat{d}}_{n}+\sum_{j=0}^{n-1}\frac{\partial \alpha_{n}}{\partial \nu {\left(t\right)}^{\left(j\right)}}\nu {\left(t\right)}^{\left(j+1\right)}\\ \; & +\sum_{j=0}^{n-1}\frac{\partial \alpha_{n}}{\partial y_{d}^{\left(j\right)}}y_{d}^{\left(j+1\right)}+\sum_{j=1}^{n-1}\frac{\partial \alpha_{n}}{\partial x_{j}}\left(x_{j+1}^{p_{j}}+\phi_{j}\left({\bar{x}}_{j}\right)+{\hat{d}}_{j}\right)+\sum_{j=1}^{n-1}\frac{\partial \alpha_{n}}{\partial {\hat{d}}_{j}}\lambda_{j}e_{j}{]}^{\frac{1}{p_{n}}}, \end{array}$$

and the derivative of $V_{n}$ can be found straightforwardly:

(42) $$\begin{array}{c} \begin{array}{c} {\dot{V}}_{n}\leq -\sum_{j=1}^{n}k_{j}s_{j}^{p-p_{j}+2}+\sum_{j=1}^{n}e_{j}^{p-p_{j}+1}{\dot{d}}_{j}-\sum_{j=1}^{n}\left(\lambda_{j}-\left(n-j+1\right)c_{j}\right)e_{j}^{p-p_{j}+2}. \end{array} \end{array}$$

**Remark 5.**  *In the whole process above, in order to counteract the crossing terms consisting of the coupling among disturbances, system states, and compensation errors, two sets of auxiliary terms, $a_{i}$ and ${\hat{a}}_{i}$, are constructed and introduced into both the virtual laws and actual control input of the back-stepping control design.*

### 3.2. Stability Analysis

So far, the design of a back-stepping control protocol has been achieved. The two conclusions areas follows.

**Theorem 1.**  *Consider the control developed by observer error system (9), corresponding system (11), and controller (41) under Assumptions 1 and 2. The closed-loop system (1) is input-to-state stable (ISS).*

**Proof of Theorem 1.**  Choose the Lyapunov function as

$$V_{n}=\sum_{i=1}^{n}\frac{s_{j}^{p-p_{j}+2}}{p-p_{j}+2}+\sum_{i=1}^{n}\frac{e_{j}^{p-p_{j}+2}}{p-p_{j}+2};$$

according to former work (42), one has

(43) $$\begin{array}{c} \begin{array}{cc} {\dot{V}}_{n}\leq & -\sum_{j=1}^{n}k_{j}s_{j}^{p-p_{j}+2}+\sum_{j=1}^{n}e_{j}^{p-p_{j}+1}{\dot{d}}_{j}-\sum_{j=1}^{n}\left(\lambda_{j}-\left(n-j+1\right)c_{j}\right)e_{j}^{p-p_{j}+2}. \end{array} \end{array}$$

In order to facilitate the following theoretical analysis, we select a constant σ,0<σ<1, and let $\lambda_{j}=\mu_{j}+\left(n-j+1\right)c_{j},\;\mu_{j}>0,\;\hat{\mu }=\min\left\{\mu_{1},\ldots ,\mu_{n}\right\}$. Then, (43) is rewritten as

(44) $$\begin{array}{c} \begin{array}{cc} {\dot{V}}_{n}\leq & -\sum_{j=1}^{n}k_{j}s_{j}^{p-p_{j}+2}+\sum_{j=1}^{n}e_{j}^{p-p_{j}+1}{\dot{d}}_{j}-\sum_{j=1}^{n}\mu_{i}e_{j}^{p-p_{j}+2}\\ \leq & -\sum_{j=1}^{n}k_{j}s_{j}^{p-p_{j}+2}+\|\dot{d}{\left\|\|e\right\|}^{p-p_{j}+1}-\hat{\mu }{\left\|e\right\|}^{p-p_{j}+2}\\ = & -\sum_{j=1}^{n}k_{j}s_{j}^{p-p_{j}+2}+\|\dot{d}{\left\|\|e\right\|}^{p-p_{j}+1}-\sigma \hat{\mu }{\left\|e\right\|}^{p-p_{j}+2}-\left(1-\sigma \right)\hat{\mu }{\left\|e\right\|}^{p-p_{j}+2}, \end{array} \end{array}$$

where $e={\left(e_{1},\ldots ,e_{n}\right)}^{T},\;\dot{d}={\left({\dot{d}}_{1},\ldots ,{\dot{d}}_{n}\right)}^{T}$.

Consider (44). It is plain that when $\left\|e\right\|\geq \frac{\|\dot{d}\|}{\hat{\mu }\sigma },$ one has

$$\begin{array}{cc} {\dot{V}}_{n}\; & \leq -\sum_{j=1}^{n}k_{j}s_{j}^{p-p_{j}+2}-\left(1-\sigma \right)\hat{\mu }{\left\|e\right\|}^{p-p_{j}+2}\\ \; & \leq -\sum_{j=1}^{n}k_{j}s_{j}^{p-p_{j}+2}-\left(1-\sigma \right)\hat{\mu }{\left\|e\right\|}^{2}. \end{array}$$

Therefore, according to Lemma 1, regarding *e* and $\dot{d}$ as state and input, respectively, the closed-loop system is input-to-state stable. Furthermore, it follows that $s_{i},\;e_{i}$ are uniformly ultimately bounded [36]. □

### 3.3. Prescribed Performance and Convergence Analysis

Next, we discuss the asymptotical output tracking of system (1) with disturbances and the prescribed performance control.

**Theorem 2.**  *Under Assumptions 1 and 2, consider the nonlinear system (1) with disturbance observer (7) and composite controller (41). Then, the following three control objectives are achieved:*

*(i)*  *the disturbance estimation error $e_{i}$ asymptotically converge to zero;*
*(ii)*  *the tracking error E(t) satisfies $\lim_{t\to \infty }E\left(t\right)=0;$*
*(iii)*  *the prescribed performance (3) is guaranteed.*

**Proof of Theorem 2.**  According to Theorem 1, regarding ${\dot{d}}_{i}\left(t\right)$ as the control input to system (1), with the help of the second condition of Assumption 1 and Lemma 2, the states satisfy

$\lim_{t\to \infty }s_{i}\left(t\right)=0,\;\lim_{t\to \infty }e_{i}\left(t\right)=0,$ which implies that

$$\lim_{t\to \infty }s_{1}\left(t\right)=\lim_{t\to \infty }\left(\frac{1}{2}\ln\frac{T\left(\chi \left(t\right)\right)+\underline{\delta }}{\bar{\delta }-T\left(\chi \left(t\right)\right)}-\frac{1}{2}\ln\frac{\underline{\delta }}{\bar{\delta }}\right)=0.$$

Then, we have $\lim_{t\to \infty }T\left(\chi \left(t\right)\right)=0;$ therefore,

$$\lim_{t\to \infty }E\left(t\right)=\lim_{t\to \infty }\nu \left(t\right)T\left(\chi \left(t\right)\right)=0.$$

In addition, since $s_{1}\left(t\right)$ is bounded, χ(t) is bounded. According to the properties of transforming function T(χ(t)) in Remark 2, $-\underline{\delta }<T\left(\chi \left(t\right)\right)<\bar{\delta },$ which means that $-\underline{\delta }\nu \left(t\right)<E\left(t\right)<\bar{\delta }\nu \left(t\right).$ Thus, the tracking error E(t) with prescribed error performance (3) is achieved. □

**Remark 6.**  *The control protocol proposed in this paper can achieve a global results for any initial conditions, and also can satisfy any performance constraints about the speed of convergence, the steady-state error, and the overshoot, which are various in practical engineering applications.*

**Remark 7.**  *In the proposed control protocol, the selection of the parameters $\underline{\delta },\;\nu \left(0\right)$ and $\bar{\delta }$ should be proper to guarantee the initial conditions of prescribed performance $-\underline{\delta }\nu \left(0\right)<E\left(0\right)<\bar{\delta }\nu \left(0\right)$. For instance, a large χ(t) will lead the tracking error E(t) to be close to its boundary, which causes a large control input u(t). However, this situation may be too strict to fit the limitations of the hardware. Reselecting the parameters $\underline{\delta },\;\nu \left(0\right)$ and $\bar{\delta }$ may be a practicable solution.*

## 4. Simulation

In order to show the practical effectiveness of the design protocol proposed in this paper, we applied it to the following second-order nonlinear system as an application and illustration:

(45) $$\left\{\begin{array}{c} \dot{x_{1}}\left(t\right)=x_{2}^{p_{1}}\left(t\right)+d_{1}\left(t\right),\\ \dot{x_{2}}\left(t\right)=u^{p_{2}}\left(t\right)-\frac{49}{10}\sin x_{1}\left(t\right)+d_{2}\left(t\right),\\ y\left(t\right)=x_{1}\left(t\right), \end{array}\right.$$

where $p_{1}=1,\;p_{2}=3,\;p=3$. Our objective was to track the expected signal $y_{d}\left(t\right)$.

In this case, consider $y_{d}\left(t\right)=0.3\sin\left(t\right)+0.2\cos\left(0.5t\right)$, and the disturbances are given as

$$d_{1}\left(t\right)=\left\{\begin{array}{c} 0,\;\;\;\;\;\;0<t<10\\ \cos(t),\;\;\;\;\;10\leq t<25,\\ 1.8,\;\;t\geq 25 \end{array}\right.$$

$$\;\;\;d_{2}\left(t\right)=\left\{\begin{array}{c} 0,\;\;\;\;\;0<t<10\\ 0.2\sin(t),\;10\leq t<25.\\ 1,\;\;\;\;\;t\geq 25 \end{array}\right.$$

In addition, let the initial condition $x_{1}\left(0\right)=0,\;x_{2}\left(0\right)=0.$ Additionally, we selected the parameters $t_{0}=1,\nu_{0}=2,\nu_{\infty }=0.1,\rho_{\infty }=0.1,\underline{\delta }=1,$ and $\bar{\delta }=2$; and $k_{1}=1,k_{2}=1,c_{1}=2,c_{2}=2$, $\lambda_{1}=5,$ and $\lambda_{2}=22$ of the prescribed performance function and the composite controller, respectively.

**Figure 1:**
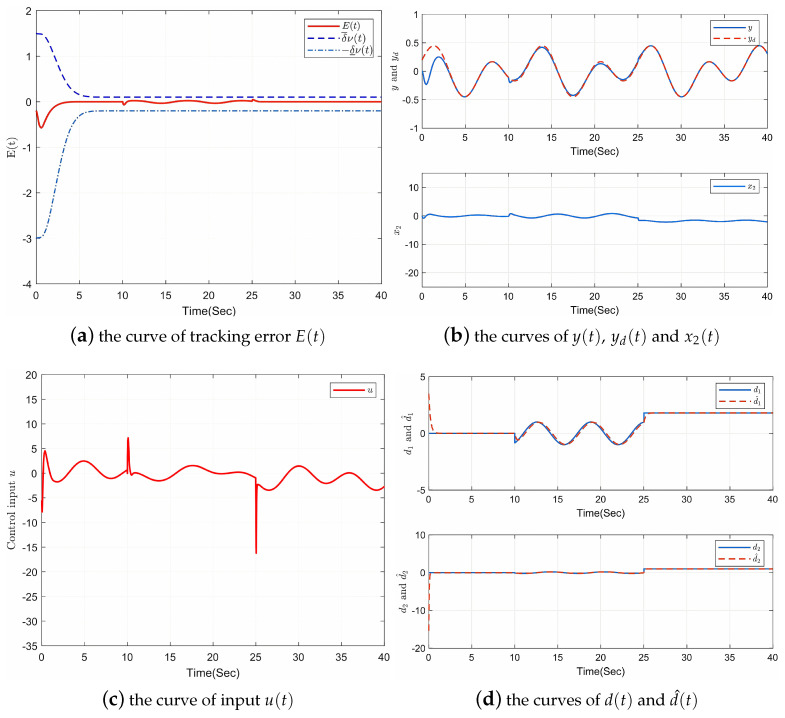
Response curves of system (45).

Figure 1 shows the simulation results. Firstly, as Figure 1a shows, the prescribed performance of tracking error E(t) can be confirmed, and disturbances have been rejected superbly by the controller, which shows the effectiveness of the proposed control protocol. Secondly, Figure 1b presents the curves of the output y(t), the given signal $y_{d}\left(t\right)$, and the state of $x_{2}\left(t\right)$, which indicates that $x_{2}$ is bounded and y(t) can fit $y_{d}\left(t\right)$ completely in less than 5 s. Thirdly, The curve of input u(t) can be seen in Figure 1c, which is also bounded. It is noted that when there are large fluctuations in disturbances from t=10 to t=25, u(t) also changes considerably at t=25 and u(t). Lastly, Figure 1d ensures the effectiveness of disturbance observer by showing that $d_{1}\left(t\right)$ and $d_{2}\left(t\right)$ can be well estimated. Therefore, it is obvious that the proposed composite controller achieves all the control objectives, which proves that it has good tracking control and anti-disturbance performance.

**Remark 8.**  *It is worth noting that the first-order case of above example can be applied to the single-link robot dynamic equation as a engineering application. The single-link robot dynamic equation proposed by Ho et al. [39] can be described as*

(46) $$\left\{\begin{array}{c} M\ddot{q}+\frac{1}{2}mgL\sin q=u,\\ y=q, \end{array}\right.$$

*where m,L, and q are the mass, the length, and the angle of the link; M=1 and g=9.8m/s denote the moment of inertia and the gravity coefficient, respectively; u is the controlling torque. Let q and $\ddot{q}$ be $x_{1}\left(t\right)$ and $x_{2}\left(t\right)$; $d_{1}\left(t\right)$ and $d_{2}\left(t\right)$ are unknown external disturbances. (10) can be written as*

(47) $$\left\{\begin{array}{c} \dot{x_{1}}\left(t\right)=x_{2}\left(t\right)+d_{1}\left(t\right),\\ \dot{x_{2}}\left(t\right)=u\left(t\right)-\frac{49}{10}mL\sin x_{1}\left(t\right)+d_{2}\left(t\right),\\ y\left(t\right)=x_{1}\left(t\right). \end{array}\right.$$

*Let m=L=1, (47) is the first-order case of (45) as*

$$\left\{\begin{array}{c} \dot{x_{1}}\left(t\right)=x_{2}\left(t\right)+d_{1}\left(t\right),\\ \dot{x_{2}}\left(t\right)=u\left(t\right)-\frac{49}{10}\sin x_{1}\left(t\right)+d_{2}\left(t\right),\\ y\left(t\right)=x_{1}\left(t\right). \end{array}\right.$$

## 5. Conclusions

In this article, the prescribed performance tracking control and anti-disturbance control problems have been solved for a class of high-order, strict-feedback systems with external disturbances. With the help of the PPC method, the DOB technique, the back-stepping method, and the technique of adding a power integrator, a novel composite controller was developed to guarantee that all states in the closed-loop system are stable and the tracking error maintains the prescribed performance throughout the evolution. In addition, the output tracking error converges to zero when the disturbances satisfy a weak assumption of boundedness. At last, a numerical simulation was presented to show the effectiveness of the theoretical result.

## Data Availability

Not applicable.
